# Assessing the role of genotype by environment interaction of winter wheat cultivars using envirotyping techniques in North China

**DOI:** 10.3389/fpls.2025.1538661

**Published:** 2025-02-11

**Authors:** Haiwang Yue, Yanbing Wang, Zhaoyang Chen, Jiashuai Zhu, Partha Pratim Behera, Pengcheng Liu, Haoxiang Yang, Jianwei Wei, Junzhou Bu, Xuwen Jiang, Wujun Ma

**Affiliations:** ^1^ Hebei Provincial Key Laboratory of Crops Drought Resistance Research, Dryland Farming Institute, Hebei Academy of Agriculture and Forestry Sciences, Hengshui, China; ^2^ Institute of Cereal and Oil Crops of Hebei Academy of Agriculture and Forestry Sciences, Shijiazhuang, China; ^3^ Faculty of Science, The University of Melbourne, Parkville, VIC, Australia; ^4^ Department of Plant Breeding and Genetics, Assam Agricultural University, Jorhat, India; ^5^ College of Agronomy, Qingdao Agricultural University, Qingdao, China; ^6^ Food Processing Engineering Technology Research and Development Center, Shandong Bairuijia Food Co., Ltd., Laizhou, China

**Keywords:** mega-environment, GGE biplot, mixed model, grain yield, envirotyping techniques

## Abstract

**Introduction:**

Winter wheat is a crucial crop extensively cultivated in northern China, where its grain yield is influenced by genetic factors (G), environmental conditions (E), and their interactions (GEI). Accurate yield estimation depends on understanding the patterns of GEI in multi-environment trials (METs).

**Methods:**

From 2014 to 2018, continuous experiments were conducted in the Heilonggang region of the North China Plain (NCP), evaluating 71 winter wheat genotypes across 16 locations over five years. Leveraging 30 years of environmental data, including 19 meteorological parameters and 6 soil physicochemical properties, the study analyzed GEI and identified four distinct mega-environments (MEs) using advanced environmental classification techniques.

**Results:**

Variance analysis of genotype-year combinations at individual locations revealed significant differences among genotypes. Furthermore, the joint analysis showed that GEI variance exceeded the variance attributed to genotypic effects alone. The Additive Main Effects and Multiplicative Interaction (AMMI) model indicates that the first three interaction principal component axes (IPCAs) account for over 70% of the GEI variance, thereby demonstrating the relevance of this model to the current study. Principal Component Analysis (PCA) across the five-year study period revealed positive correlations between grain yield and vapor pressure deficit (VPD), evapotranspiration potential (ETP), temperature range (TRANGE), available soil water (ASKSW), and sunshine duration. Conversely, negative correlations were observed with relative humidity at 2 meters (RH2M), total precipitation (PRECTOT), potential evapotranspiration (PETP), and dew point temperature at 2 meters (T2MDEW). Among the meteorological and soil variables, minimum temperature (TMIN), fruiting rate (FRUE), temperature at 2 meters (T2M), and clay content (CLAY) emerged as the most significant contributors to yield variation during the study period. Based on GGE biplot analysis, superior genotypes were identified for their respective regions: JM196, WN4176, and HN6119 in 2014; ZX4899, H9966, and LM22 in 2015; BM7, KN8162, and KM3 in 2016; HH14-4019, HM15-1, and HH1603 in 2017; and S14-6111 and JM5172 in 2018. Feixiang and Shenzhou were identified as the most discriminative and representative locations.

**Discussion:**

These findings provide a scientific basis for optimizing winter wheat cultivation strategies in northern regions. Based on long-term data from the North China Plain, future work can further validate their applicability in other regions.

## 1 Introduction

Wheat (*Triticum aestivum* L.) is one of the world’s most important food crops, with a long history of cultivation that has provided humanity with essential food and by-products, such as flour (Senapati et al., 2022; Bayissa et al., 2023). Driven by rapid population growth and rising incomes, global wheat demand is expected to increase significantly, particularly in developing countries (Roostaei et al., 2021). China is the largest producer and consumer of wheat, maintaining its position as the world’s top wheat producer in 2022, with an output of 138 million tons—17% of the global production (Wang et al., 2020, 2024; Yi et al., 2024). The wheat planting area occupies approximately 23 million hectares in China, accounting for 22%–30% of the country’s total arable land and 22%–27% of the total food crop area (Zhang et al., 2024).

Wheat is categorized into winter wheat and spring wheat based on the sowing time, with winter wheat dominating production in China. Over 80% of China’s wheat production is attributed to winter wheat (Feng et al., 2024). Wheat yield is influenced by genotype (G), environmental (E) factors, and genotype–environment interactions (GEI). These interactions complicate the selection of stable high-yielding genotypes (Le Gouis et al., 2020; Mitura et al., 2023). In multi-environment trials (METs), much of the variation in yield is caused by E and GEI, making them critical factors when breeding and recommending wheat genotypes in different regions (Crespo-Herrera et al., 2018; Saeidnia et al., 2023). Stability analysis is essential for identifying genotypes that consistently perform well across diverse environments as well as those suited to specific locations (González-Barrios et al., 2019). These analyses employ various methods, including parametric and non-parametric approaches, to evaluate genotype stability under changing environmental conditions (Vaezi et al., 2018; Pour-Aboughadareh et al., 2019; Vaezi et al., 2019; Yue et al., 2022a).

Envirotyping, which uses environmental data to model how crops grow in specific conditions, has become increasingly feasible with advancements in geographic information systems (GIS) and environmental big data. By characterizing the environment during the crop growth period, researchers can identify key factors affecting yield and adaptation (Costa-Neto et al., 2020; Resende et al., 2021). The variation in genotype responses to environmental gradients during the growing season resulted in GEI. A “mega-environment” (ME) is a group of regions with similar environmental conditions, where a specific genotype consistently performs the best, with minimal crossover interactions. Repeatable GEI can be addressed by breeding genotypes tailored to specific MEs, while non-repeatable GEI can be managed through targeted selection within an ME (Hassani et al., 2018; Shahriari et al., 2018).

Climate variability, such as changes in rainfall and temperature, poses significant challenges to global wheat production, and increases yield instability and food insecurity (Toreti et al., 2019; Raimondo et al., 2021). To mitigate these risks, it is crucial to evaluate wheat performance over multiple years and locations. Methods such as the Additive Main Effect and Multiplicative Interaction (AMMI) and Genotype Plus Genotype-by-Environment (GGE) biplots are commonly used to analyze GEI. GGE biplots are particularly useful for identifying MEs, ranking genotypes, and selecting environments for testing (Mohammadi et al., 2018; Singh et al., 2019; Bishwas et al., 2021). More recently, the use of linear mixed-effects models, such as Best Linear Unbiased Prediction (BLUP), has been shown to improve the predictive accuracy. Stability metrics, such as the Weighted Average Absolute Scores of BLUPs (WAASBs) and WAASBY indices, allow researchers to simultaneously evaluate both performance and stability (Olivoto et al., 2019a; Rajput et al., 2021).

In China, wheat cultivation zones have traditionally been based on agroclimatic regions. However, systematic METs are required to better identify MEs and recommend optimal genotypes (Liu et al., 2021). This study aimed to map the impact of environmental factors on wheat yield, understand GEI, and identify MEs by integrating environmental, genotype, and interaction effects. These efforts will help to select wheat genotypes with high yield, stability, and adaptability, ensuring sustainable production across diverse regions.

## 2 Materials and methods

### 2.1 Plant materials, locations, and experimental design

**Figure 1:**
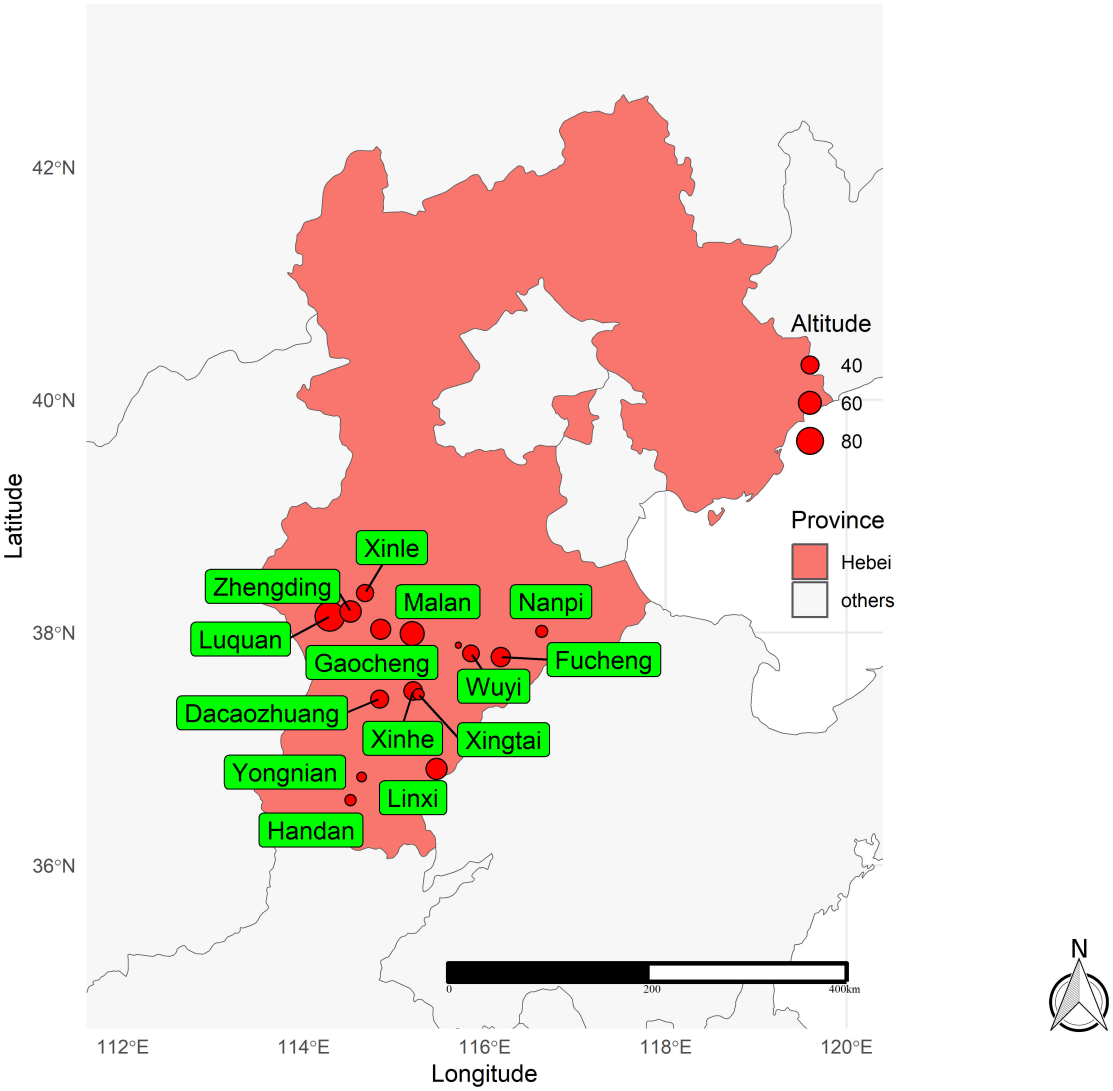
Locations of the 16 locations used in this study in the 2014–2018 crop seasons in Hebei province of China.

From 2014 to 2018, 51 fields were located in 16 different locations in the Hebei Province of China (**Table 1**, **Figure 1**). According to the annual trial results, new genotypes were added to the evaluation plan every year. A total of 14, 13, 14, 16, and 14 winter wheat genotypes were evaluated in 2014, 2015, 2016, 2017, and 2018, respectively. Information on the winter wheat genotypes evaluated each year is shown in **Supplementary Tables S1–S5**. A randomized complete block design (RCBD) was adopted with three replications in a plot of size 13.33 m^2^. The agronomic measures during the experiment were based on local field management, and the grain yield was measured in kg ha^−1^, with a correction of 13%.

**Table 1:** Basic information of the 51 environments used in this research during 2014–2018.

Code	Environment	Year	Sowing data
1	Yongnian	2014	06/10/2014
2	Yongnian	2015	09/10/2015
3	Yongnian	2016	08/10/2016
4	Yongnian	2017	09/10/2017
5	Yongnian	2018	08/10/2018
6	Handan	2014	09/10/2014
7	Handan	2015	05/10/2015
8	Handan	2017	02/10/2017
9	Handan	2018	02/10/2018
10	Feixiang	2014	10/10/2014
11	Feixiang	2015	12/10/2015
12	Shenzhou	2014	12/10/2014
13	Shenzhou	2015	10/10/2015
14	Shenzhou	2016	09/10/2016
15	Shenzhou	2017	10/10/2017
16	Shenzhou	2018	06/10/2018
17	Gaocheng	2014	10/10/2014
18	Gaocheng	2015	08/10/2015
19	Gaocheng	2016	09/10/2016
20	Gaocheng	2017	11/10/2017
21	Gaocheng	2018	12/10/2018
22	Luquan	2014	13/10/2014
23	Luquan	2015	11/10/2015
24	Luquan	2017	15/10/2017
25	Luquan	2018	11/10/2018
26	Malan	2014	10/10/2014
27	Malan	2015	12/10/2015
28	Malan	2017	13/10/2017
29	Malan	2018	11/10/2018
30	Wuyi	2014	13/10/2014
31	Wuyi	2015	15/10/2015
32	Wuyi	2018	12/10/2018
33	Xinhe	2014	15/10/2014
34	Xinhe	2015	14/10/2015
35	Xinhe	2016	16/10/2016
36	Xingtai	2014	08/10/2014
37	Xingtai	2015	10/10/2015
38	Xingtai	2016	11/10/2016
39	Xingtai	2017	09/10/2017
40	Xingtai	2018	10/10/2018
41	Zhengding	2014	15/10/2014
42	Zhengding	2016	13/10/2016
43	Nanpi	2015	12/10/2015
44	Nanpi	2016	14/10/2016
45	Nanpi	2017	12/10/2017
46	Nanpi	2018	15/10/2018
47	Xinle	2015	14/10/2015
48	Xinle	2017	16/10/2017
49	Linxi	2017	15/10/2017
50	Dacaozhuang	2018	14/10/2018
51	Fucheng	2016	15/10/2016

### 2.2 Classification of mega-environments based on 30 years of meteorological factors and soil data

Firstly, 19 meteorological factors from 16 locations from 1989 to 2019 were collected using the R package EnvRtype (Costa-Neto et al., 2021). To better classify mega-environments (MEs), we filtered the data for 19 meteorological factors each year and only screened for meteorological data covering the winter wheat growing season (October to June). In addition, data for six local soil chemical factors were obtained using the SoilType package (Fritsche-Neto, 2023). A dataset containing 25 environmental covariates (ECs) is listed in **Table 2**. The 25 ECs were used to construct an envirotype-covariable matrix (W) that further computed the environmental affinities W_matrix using functions in the EnvRtype package. With a nine-month period (October to June) to represent the temporal variation of the crop growth period, 6,750 (30 years × 25 ECs × 9 intervals) variables were obtained, and the envirotype covariable matrix (25 environmental rows × 6,750 climatic variables’ columns) was used to calculate the enviromic kernel (KE) using the following formula:

**Table 2:** Details of the 25 environmental covariables (ECs) used in this study.

Source	Environmental factor	Unit
Nasa POWER^a^	All sky insolation incident on a horizontal surface ASKSW	MJ m^−2^ d^−1^
	Downward thermal infrared (longwave) radiative flux ASKLW	MJ m^−2^ d^−1^
	Extraterrestrial radiation RTA	MJ m^−2^ d^−1^
	Wind speed at 2 m above the surface of the earth WS2M	m s^−1^
	Minimum air temperature at 2 above the surface of the earth TMIN	°C d ^−1^
	Average air temperature at 2 above the surface of the earth T2M	°C d ^−1^
	Maximum air temperature at 2 above the surface of the earth TMAX	°C d ^−1^
	Dew-point temperature at 2 m above the surface of the earth T2MDEW	°C d ^−1^
	Relative air humidity at 2 above the surface of the earth RH2M	%
	Rainfall precipitation PRECTOT	mm d ^−1^
Calculated^b^	Temperature range TRANGE	°C d^−1^
	Potential evapotranspiration ETP	mm d^−1^
	Deficit by precipitation PETP	mm d^−1^
	Vapor pressure deficit VPD	kPa d^−1^
	Slope of saturation vapor pressure curve SPV	kPa °C d^−1^
	Effect of temperature on radiation-use efficiency FRUE	from 0 to 1
	Growing degree day GDD	°C d^−1^
	Actual duration of sunshine n	h
	Daylight hours N	h
Soil covariates	Clay total CLAY	g/100 g
	Sand total SAND	g/100 g
	Silt total SILT	g/100 g
	pH H2O PHAQ	
	Organic carbon ORGC	g/kg
	Total nitrogen (N) NITKJD	g/kg

^a^represents the meteorological data obtained directly from NASA orbital sensors (Sparks, 2018), ^b^represents the data was calculated from Allen et al. (1998) and Soltani and Sinclair (2012).


(1) $$\;\;\;K_{\text{E}}=\;\frac{WW^{\prime }}{\frac{trace\left(WW^{\prime }\right)}{nrow\left(W\right)}}$$

where *K_E_* is the environmental similarity kernel based on environment “omics” and W is the envirotype matrix. To identify MEs, hierarchical clustering (average linkage method) was applied to the *K_E_*.

Finally, to show the correlations between the 25 environmental variables, principal component analysis (PCA) was performed by creating a bidirectional table containing environmental variables using the ‘fviz_pca_biplot()’ function from the R package factoextra (Kassambara and Mundt, 2017).

### 2.3 Stability analysis

#### 2.3.1 Additive main effects and multiplicative interaction analysis

The additive main effects and multiplicative interaction (AMMI) model was used to examine the grain yield of the evaluated genotypes. The AMMI model integrates the standard analysis of variance (ANOVA) and principal component axis (PCA) to determine the interactive principal component axis (IPCA) and calculate stability parameters. The AMMI model proposed by Gauch (1988) is given as:


(2) $$Y_{ge}=\mu +\alpha_{g}+\beta_{e}+\sum_{n=1}^{k}\lambda_{n}\delta_{gn}\gamma_{en}+\rho_{ge}$$

where $Y_{ge}$ is the target trait yield of the *g*th genotype in the *e*th environment; *μ* represents the overall average; $\alpha_{g}$ represents the *g*th genotype effect; $\beta_{e}$ represents the *e*th environmental effect; $\lambda_{n}$ represents the *n*th principal component axis (PCA) singular value; $\delta_{gn}$ and $\gamma_{en}$ are the characteristic vector values of genotype *g*, environment *e* and component *n*, respectively; $\rho_{ge}$ is the residual; and *k* is the number of main component axes (PCA).

#### 2.3.2 The best linear unbiased prediction model for multi-environment trials

As a well-known linear model with interaction, the best linear unbiased prediction (BLUP) is often used to analyze METs data, as described by Piepho et al. (2008), as follows:


(3) $$y_{ijk}=\mu +\alpha_{i}+\tau_{j}+\alpha \tau_{ij}+\gamma_{jk}+\epsilon_{ijk}$$

where $y_{ijk}$ is the grain yield observed in the *k*th block of the *i*th genotype in the *j*th environment; μ is the grand mean; $\alpha_{i}$ and $\tau_{j}$ are the effect of the *i*th genotype and *j*th environment, respectively; $\alpha \tau_{ij}$ is the interaction effect of the *i*th genotype with the *j*th environment; $\gamma_{jk}$ is the effect of the *k*th block within the *j*th environment; and $\epsilon_{ijk}$ is the error term.

#### 2.3.3 Cross-validation procedure

Cross-validation was performed to determine the best model and evaluate the efficiency of the AMMI and BLUP models. The raw dataset is divided into two parts: training and validation. The training dataset had N − 1 replications, i.e., two replications, whereas the validation dataset had only one replication. The root-mean-square prediction difference (RMSPD) value was used to select and compare the AMMI and BLUP models. The smaller the RMSPD value, the more accurate the model prediction (Gauch, 2013).

#### 2.3.4 Estimation of stability indexes

The following stability indexes were estimated based on the AMMI and BLUP analyses. The stability indexes based on AMMI analysis were the AMMI Stability Index (ASI), AMMI Stability Value (ASV), Modified AMMI Stability Index (MASI), Modified AMMI Stability Value (MASV), Simultaneous Selection Index (SSI), Sums of the Averages of the Squared Eigenvector Values (EV), Annicchiarico’s D Parameter Values (DA), Zhang’s D Parameter (DZ), Sums of the Absolute Value of the IPC Scores (SIPC), Absolute Value of the Relative Contribution of IPCs to the Interaction (ZA), and Weighted Average of Absolute Scores (WAAS). The BLUP values of the evaluated winter wheat genotypes (**Supplementary Figure S4**) were used in the estimation of indexes *viz.*, the relative performance of the genotypic values (RPGV), the harmonic mean of genotypic values (HMGV), and the harmonic mean of the relative performance of genotypic values (HMRPGV) (Ajay et al., 2020; Anuradha et al., 2022).

To allow weighting between grain yield and stability of winter wheat genotypes, a new superiority index, WAASBY (Weighted Average of Absolute Scores of BLUP (WAASB) and yield) was used. The best genotype was determined by rescaling and weighing grain yield (GY) and stability index (WAASB) (Olivoto et al., 2019b). The weighting values for the WAASB and GY were 50 and 50, respectively, which gave equal weights to both metrics. Various stability indexes were obtained using the R metan package (Olivoto and Lúcio, 2020).

#### 2.3.5 GGE biplot analysis

The yield data were analyzed using GGE biplot analysis. GGE biplots have the following functions: a) can visually present the mean genotypic performance across environments; b) can study the “which-won-where” mode across the mega-environments (MEs); c) can evaluate the discrimination and representativeness of the testing environments; d) can compare the target genotype with the ideal genotype. Yan (2001) proposed the following GGE biplot model:


(4) $$Y_{ij}=\mu +\beta_{j}+\lambda_{1}\xi_{i1}\eta_{j1}+\lambda_{2}\xi_{i2}\eta_{j2}+\epsilon_{ij}$$

where $Y_{ij}$ is the expected grain yield of genotype *i* in environment *j*; μ is the grand mean; $\beta_{j}$ is the main influence value of environment *j*; $\lambda_{1}$ and $\lambda_{2}$ are the singular values of the first and second principal components (PC1 and PC2), respectively, $\xi_{i1}$ and $\xi_{i2}$ are the feature vectors of genotype *i* for PC1 and PC2, respectively, $\eta_{j1}$ and $\eta_{j2}$ represent the special vectors of environment *j* for PC1 and PC2, respectively, and $\epsilon_{ij}$ is the unexplained residues of genotype *i* in environment *j*. The GGE biplot analysis and mapping was done by the R metan package.

### 2.4 Statistical software

All statistical analyses of the raw data involved in this study were performed using R software 4.3.1 (R Core Team, 2022) with the packages mentioned previously.

## 3 Results

### 3.1 Envirotyping

**Figure 2:**
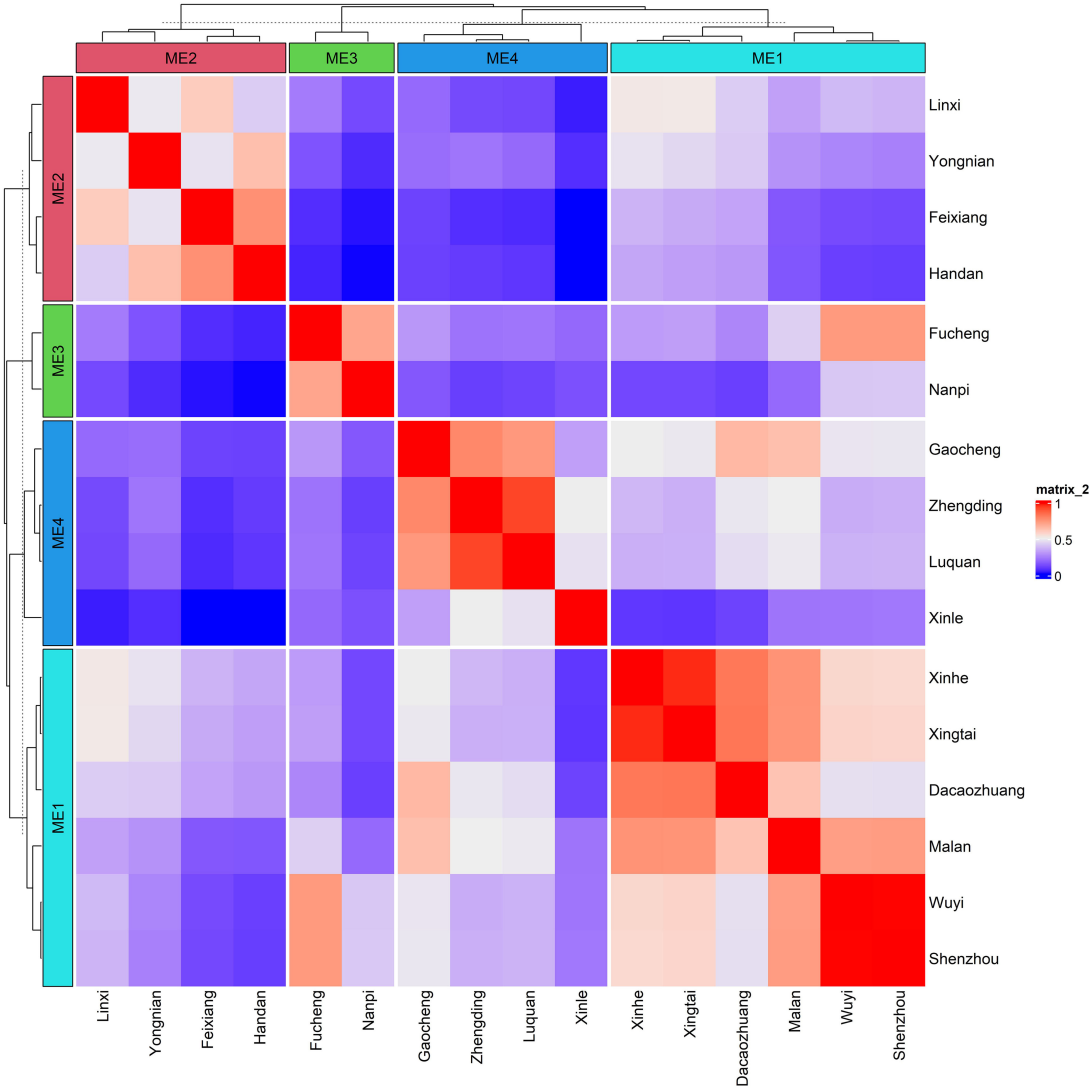
The heat map depicting the delineated mega-environments considering the environmental similarity based on 30 years of climate information on 19 meteorological covariates and six soil factors.

Based on 30 years (1989–2019) of climate and soil information, four mega-environments (MEs) were identified using similarity analysis of 25 environmental covariates (ECs), including 19 meteorological factors and six soil physicochemical factors. ME1 comprised locations—Shenzhou, Wuyi, Malan, Dacaozhuang, Xingtai, and Xinhe. ME2 includes locations such as Linxi, Yongnian, Feixiang, and Handan. Nanpi and Fucheng were included in ME3, whereas Gaocheng, Zhengding, Luquan, and Xinle were included in ME4 (**Figure 2**). These MEs exhibited geographic proximity, with the first two principal components accounting for 68.8% of location variability, indicating significant differences in environmental variables across environments (**Supplementary Figure S1**).

**Figure 3:**
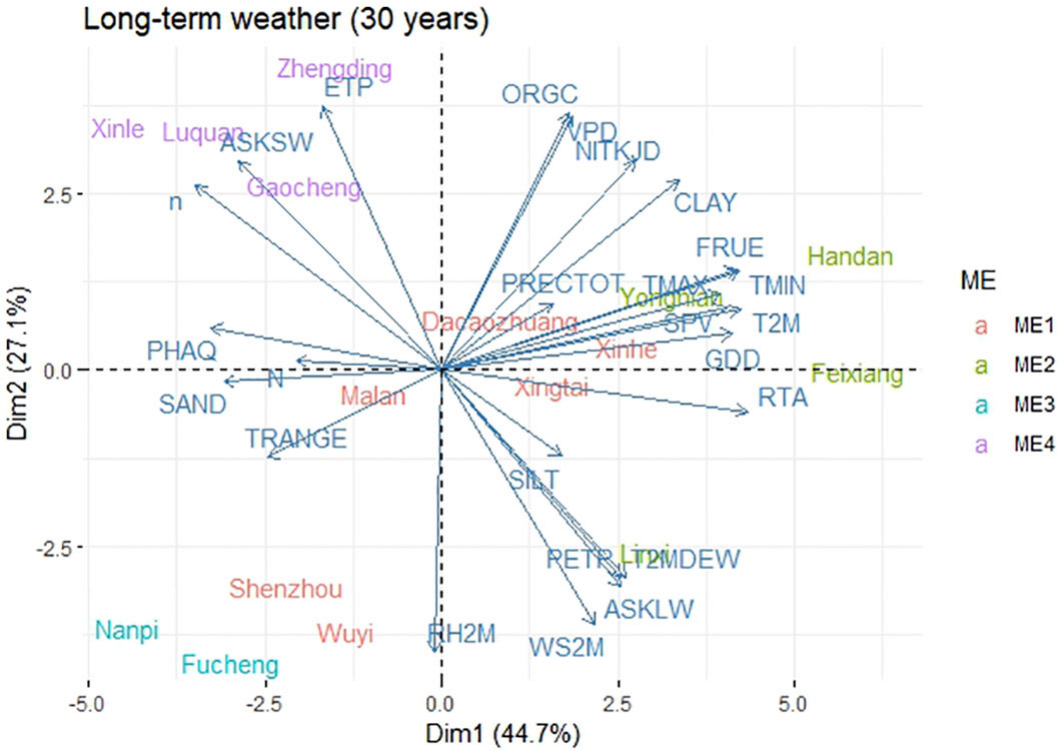
Biplot for the principal component analysis between environmental variables during 1989-2019.

**Figure 4:**
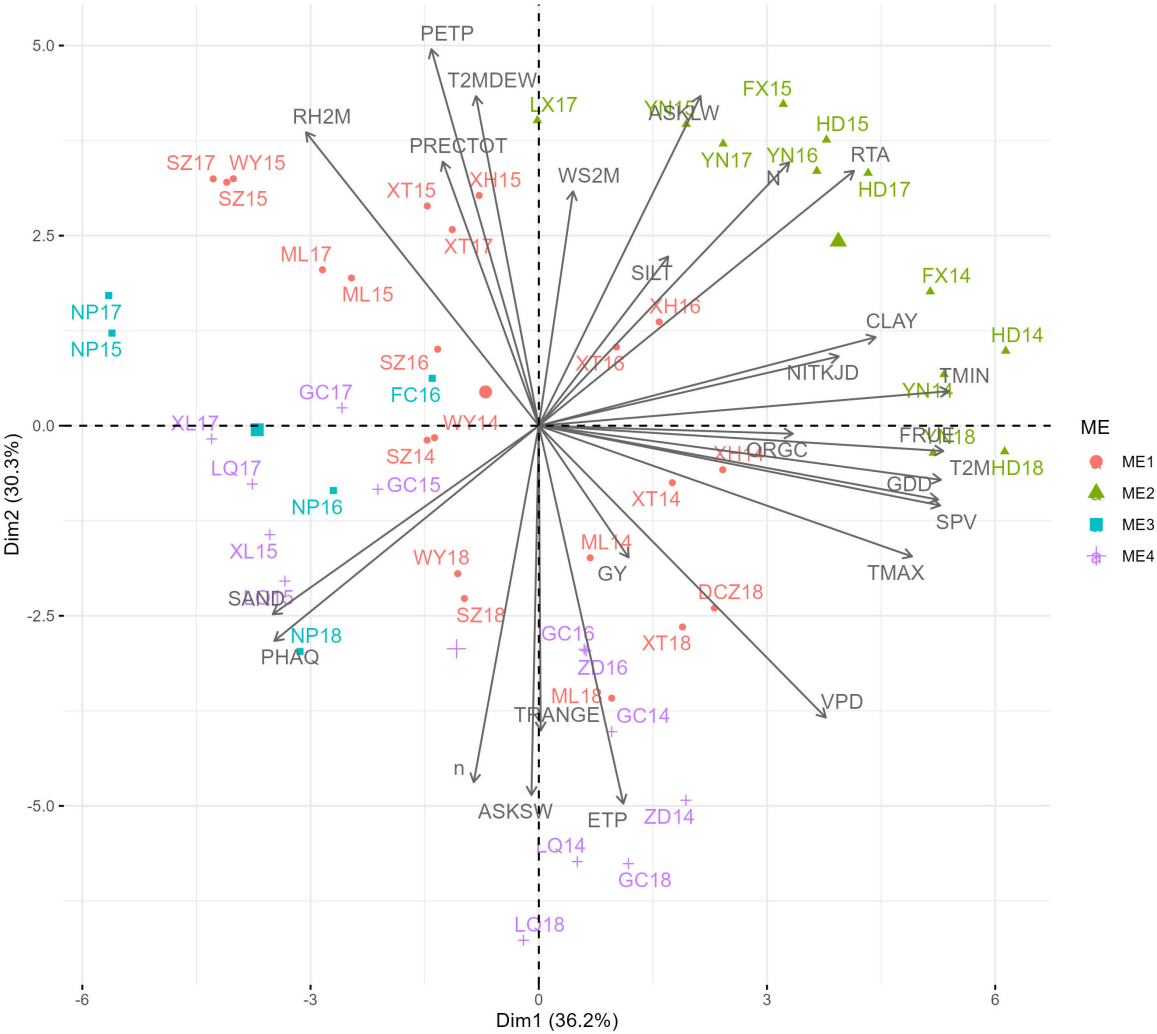
Biplot for the principal component analysis between environmental variables in the trials during 2014-2018.

Principal component analysis based on 30-year climate data and soil composition information revealed that ME1 had higher TRANGE, PRECTOT, GDD, N, SAND, PHAQ, and SILT values. ME2 had higher RTA, WS2M, FRUE, SPV, TMAX, ASKLW, and PETP. ME3 had lower climate and soil variables, namely, VPD, ORGC, NITKJD, and CLAY, and ME4 was associated with higher ETP, ASKSW, and n (**Figure 3**). RTA, T2M, TMIN, FRUE, and GDD were the climatic variables that contributed the most to the environmental scores (**Supplementary Figure S2**). During the 2014–2018 METs, grain yield (GY) was found to be positively correlated with environmental variables such as ETP, VPD, TMAX, SPV, GDD, T2M, FRUE, and ORGC, and negatively correlated with environmental factors such as RH2M, PRECTOT, PETP, and T2MDEW (**Figure 4**). This variation was predominantly attributed to TMIN, FRUE, T2M, SPV, and GDD during 2014–2018 (**Supplementary Figure S3**).

### 3.2 Combined analysis of variance and AMMI analysis

The combined analysis of variance for each year showed that genotype (G), environment (E), and GE were all highly significant (*P* ≤0.001) for grain yield at all locations (**Table 3**). Among the sources of variation, the environmental effect recorded the highest sum of squares, indicating that the highest degree of variation was 47.72%, 66.82%, 70.59%, 68.28%, and 69.26% in all five years, respectively. The proportions of GE interactions accounted for 15.54%, 9.51%, 7.76%, 9.70%, and 10.00%, respectively. From 2014 to 2018, genotypic main effects accounted for 12.64%, 5.46%, 3.24%, 3.96%, and 4.99% of the total variation. During 2014–2018, the proportion of total variation recorded by GE interactions was greater than that of the genotype, indicating its importance for variety performance across various locations.

**Table 3:** Combined analysis of variance for trials from 2014 to 2018.

Sources	2014			2015			2016			2017			2018		
	Df	Mean square	VE%	Df	Mean square	VE%	Df	Mean square	VE%	Df	Mean square	VE%	Df	Mean square	VE%
Environment (E)	10	22,118,160^***^	47.72	11	38,646,121^***^	66.82	7	65,175,625^***^	70.59	9	20,360,237^***^	68.28	9	56342055^***^	69.26
Rep (Env)	22	348,510^***^		24	385,746^***^		16	1,265,350^***^		20	143,058^**^		20	114972	
Genotype (G)	13	4,534,367^***^	12.64	12	2,890,962^***^	5.46	13	1,605,581^***^	3.24	15	704,175^***^	3.96	13	2809911^***^	4.99
GE Interaction	130	556,753^***^	15.54	132	458,689^***^	9.51	91	551,094^***^	7.76	135	192,429^***^	9.70	117	625989^***^	10.00
Residuals	286	115,575		288	158,202		208	234,316		300	64,933		260	153552	
CV (%)	4.13			4.61			5.94			4.04			4.88		
Overall mean (kg/ha)	8,226.31			8,634.40			8,153.96			6,309.98			8030.49		

Df, degree of freedom; **, significant at *P* ≤0.01; ***, highly significant at *P* ≤0.001; VE%, percentage of variance explained.

The AMMI analysis results for all five years are presented in **Table 4**. The GE interaction matrix was partitioned to form a multiplicative component. The first three IPCAs (interaction principal component axes) obtained by singular value decomposition of GE interactions were statistically significant (*p <*0.01) using the F-test. IPCA1 accounted for 27.7%, 32.0%, 33.9%, 44.2%, and 55.5% of total GE interactions between 2014 and 2018, respectively.

**Table 4:** AMMI analysis of variance of grain yield for trials from 2014 to 2018.

Sources	2014			2015			2016			2017			2018		
	Df	F value	Proportion %	Df	F value	Proportion %	Df	F value	Proportion %	Df	F value	Proportion %	Df	F value	Proportion %
Environment (E)	10	63.46^***^		11	100.19^***^		7	51.51^***^		9	142.32^***^		9	490.05^***^	
Rep (Env)	22	3.02^**^		24	2.44^***^		16	5.40^***^		20	2.20^**^		20	0.75	
Genotype (G)	13	39.23^***^		12	18.27^***^		13	6.85^***^		15	10.84^***^		13	18.30^***^	
GE Interaction	130	4.82^***^		132	2.9^***^		91	2.35^***^		135	2.96^***^		117	4.08^***^	
IPCA1	22	7.90^***^	27.7	22	5.57^***^	32.0	19	3.82^***^	33.9	23	7.68^***^	44.2	21	12.61^***^	55.5
IPCA2	20	6.67^***^	21.3	20	3.84^***^	20.0	17	3.24^***^	25.8	21	4.17^**^*	21.9	19	5.17^***^	20.6
IPCA3	18	6.41^***^	18.4	18	3.80^***^	17.9	15	2.43^**^	17.0	19	3.17^***^	15.1	17	2.18^**^	7.8
Residuals	286	–	–	288	–	–	208	–	–	300	–	–	260	–	–
Total	591	–	–	599	–	–	426	–	–	614	–	–	536	–	–

Df, degree of freedom; **, significant at *P* ≤0.01; ***, highly significant at *P* ≤0.001; IPCA, interaction principal component axis.

### 3.3 Model comparison between BLUP and AMMI families

**Figure 5:**
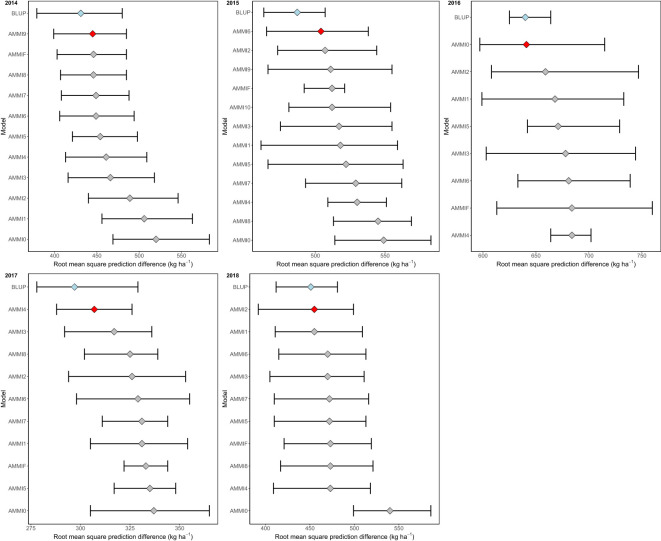
The distribution of 1,000 estimates of root mean square prediction difference (RMSPD) was visualized by boxplot to compare the prediction accuracy of BLUP and AMMI families.

The evaluation identified the optimal models for the period 2014–2018. Based on our multi-year datasets showing various genotype-environment interaction (GEI) patterns, our analysis indicated that the Best Linear Unbiased Prediction (BLUP) model provided the most accurate predictions. Furthermore, we observed that AMMI9, AMMI6, AMMI0, AMMI4, and AMMI2 demonstrated the highest accuracy among the AMMI models during the period 2014–2018 (**Figure 5**).

### 3.4 Stability indices

**Figure 6:**
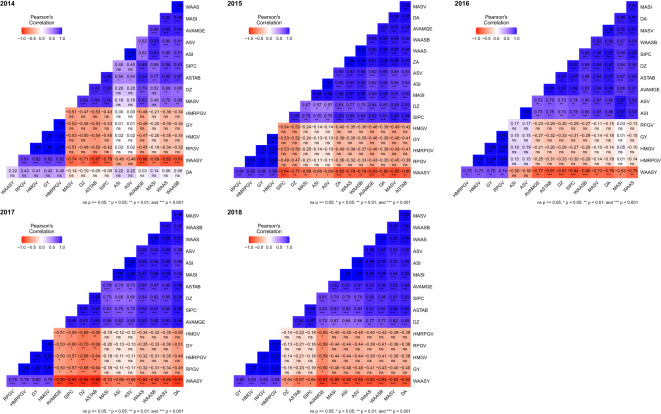
The heatmap showing the correlation between stability indices based on AMMI and BLUP along with grain yield. ns, *p* ≥0.05; *p <0.05; **p <0.01; ***p <0.001.

A heat map (**Figure 6**) was generated to compare various stability indices derived from the AMMI, BLUP, and WASSY methods based on their rankings (**Supplementary Tables S6**, **S7**). The rank correlation analysis of these indices demonstrated that the BLUP and AMMI indices formed two separate clusters with a strong correlation observed within each cluster, while correlations between clusters were weaker.

During the period from 2014 to 2018, the correlation between AMMI indices and grain yield varied between −0.53 to 0.42, −0.53 to −0.13, −0.33 to 0.15, −0.70 to −0.13, and −0.60 to −0.15, respectively, for each respective year, whereas the correlation of the BLUP index with grain yield was 1.00^***^ to 1.00^***^, 1.00^***^ to 1.00^***^, 0.99^***^ to 1.00^***^, 1.00^***^ to 1.00^***^, and 1.00^***^ to 1.00^***^ across the same time frame. Overall, this suggests that the BLUP indices exhibited a high correlation with grain yield, whereas the AMMI indices displayed a low or negative correlation.

The WAASY index was significantly and positively correlated with grain yield and BLUP indices. This suggests that the WAASY index considers both grain yield and GE interactions when identifying the best genotypes. The correlation between WAASY index and AMMI index in 2014–2018 was −0.48 to −0.86^***^, −0.60^*^ to −0.85^***^, −0.50 to 0.84^***^, −0.68^**^ to −0.94^***^ and −0.54^*^ to −0.91^***^, respectively, while the correlation with BLUP index was 0.81^***^ to 0.82^***^, 0.84^***^ to 0.86^***^, 0.73^***^ to 0.75^***^, 0.78^***^ to 0.79^***^ and 0.83^***^ to 0.84^***^, respectively.

### 3.5 GGE biplot analysis

**Figure 7:**
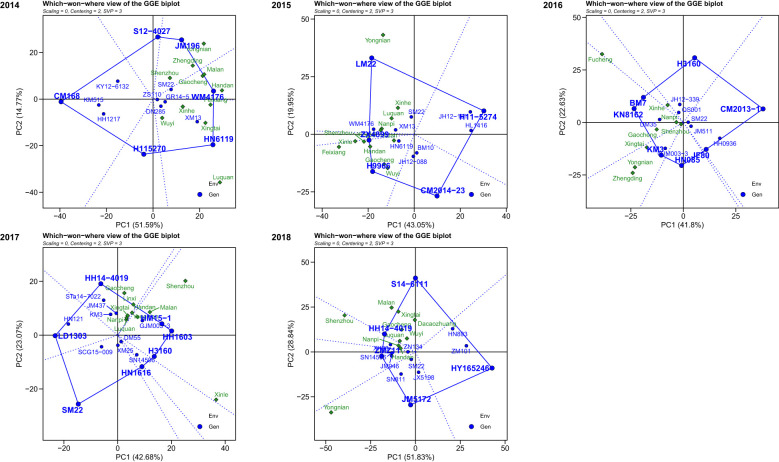
The which-won-where view of the GGE biplot for multi-environment trials from 2014 to 2018.

According to the GGE biplot analysis, the first two PCs (principal components) explained 66.36%, 62.60%, 64.43%, 65.75%, and 80.67% of the total genotype and GE variation from 2014 to 2018, respectively. The winning genotype at each location was identified using the which-won-where view of the GGE biplot. During 2014, genotypes JM196 exhibited high yield in locations Shenzhou, Zhengding, and Yongnian to become the champion genotype, while genotypes WM4176 and HN6119 were identified as superior genotypes in the remaining locations. During 2015, genotypes ZX4899 and H9966 became the general winners across six locations including Xinle, Feixiang, Shenzhou, Handan, Gaocheng, and Wuyi, and genotype LM22 became the universal winner across the remaining five locations. During 2016, genotypes BM7, KN8162, and KM3 were the champion genotypes on locations Fucheng and Xinhe, locations Nanpi and Gaocheng, locations Zhengding, Yongnian, Xingtai, and Shenzhou, respectively. During 2017, the champion genotype across locations Gaocheng, Linxi, Handan, Xingtai, Nanpi, and Luquan was HH14-4019, while the genotypes HM15-1 and HH1603 performed well in the locations Yongnian, Malan, Shenzhou, and Xinle as the universal winner. During 2018, the genotype S14-6111 had broad adaptability in the locations Malan, Xingtai, Dacaozhuang, Gaocheng, Wuyi, and Luquan as the universal genotype, while JM5172 performed best in the location Yongnian (**Figure 7**).

**Figure 8:**
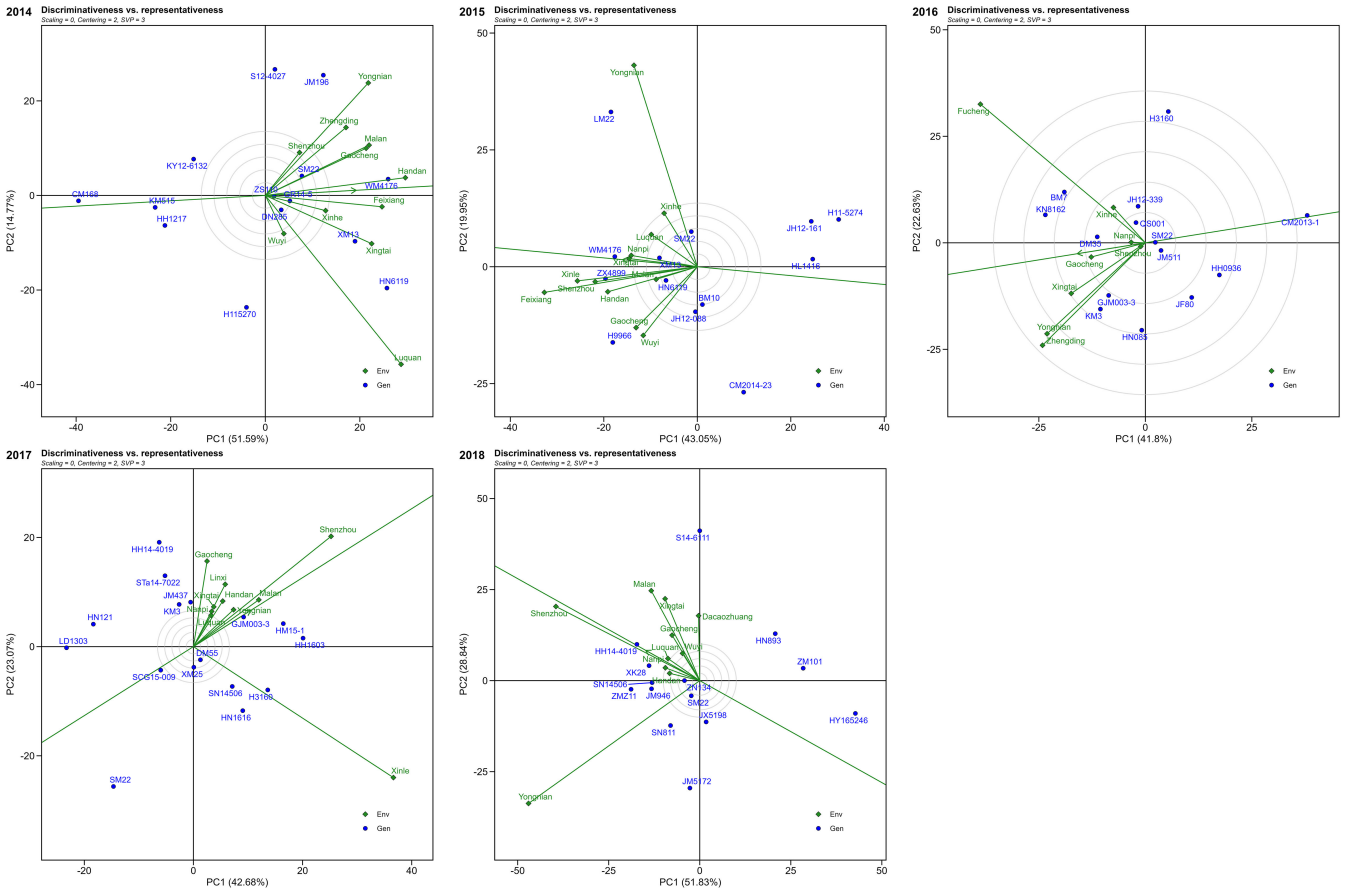
The discriminativeness vs. representativeness view of the GGE biplot for multi-environment trials from 2014 to 2018.

Based on the discriminative and representative view of GGE biplot, during 2014, Yongnian, Malan, Gaocheng, Handan, Feixiang, and Luquan were the most discriminative locations. Moreover, the Handan and Feixiang locations were more representative. Thus, Handan and Feixiang were locations with both discrimination and representativeness. In 2015, Yongnian, Xinle, and Feixiang were the most discriminative locations, and Nanpi and Xingtai were more representative. In 2016, the highest discriminative locations were Fucheng, Yongnian, Xingtai, and Zhengding, whereas the representative location was Gaocheng. During 2017, locations Shenzhou and Xinle were higher discriminative, while Yongnian, Malan, Shenzhou, and Xingtai showed good representative ability among the test locations. Thus, Shenzhou was the most representative and best discriminating location for evaluating winter wheat. Likewise, during 2018, Shenzhou, Yongnian, Malan, and Xingtai were the higher discriminative locations, while Shenzhou, Luquan, Nanpi, and Handan showed good representative ability. Shenzhou was the location with higher discriminative and good representative (**Figure 8**).

## 4 Discussion

### 4.1 Impact of genotype–environment interactions on winter wheat yield

The results of this study confirm that GEI plays a significant role in determining winter wheat yield. The findings revealed that environmental factors, especially temperature, relative humidity, and vapor pressure deficit (VPD), significantly affected genotypic performance. These interactions are particularly pronounced in multi-year and multi-location trials, where the variability of the GEI exceeds that of genotype effects alone. This result aligns with those of previous studies (Özdoğan, 2011; Semenov et al., 2014; Bajwa et al., 2020), highlighting that environmental factors, especially those linked to climate change, cannot be overlooked in wheat production. The North China Plain (NCP), a region with dynamic agricultural conditions, serves as a prime example of how the GEI shapes genotype performance (Xiao et al., 2020; Zhang et al., 2020). Understanding the role of environmental changes in wheat growth is crucial for the selection of genotypes with superior adaptability. In this context, selecting genotypes with stable and high yield potential across various environments is paramount, as this can directly improve productivity in regions affected by climate variability (Nataraj et al., 2024).

### 4.2 Environmental factors and their influence on wheat growth

The study revealed that warmer conditions, especially in locations such as Dacaozhuang, Xinhe, and Xingtai, led to elevated temperatures (T2M, TMAX, and TMIN), reduced relative humidity, and consequently, higher VPD. These conditions result in increased evapotranspiration (ETP), especially in Xinhe, suggesting that water loss through evapotranspiration is more pronounced in warmer environments. This positive correlation between environmental temperature and ETP supports the hypothesis that environmental warming increases the challenges associated with water loss in wheat production (Casagrande et al., 2024). These findings underscore the need for climate-resilient wheat genotypes that can withstand environmental stresses. Genotypes that exhibit stable yields under varying temperature and relative humidity are essential for maintaining productivity in the face of climate change. Focusing on these environmental factors when selecting drought-resistant or heat-tolerant traits could improve the resilience of future wheat crops (Wang et al., 2023).

### 4.3 Statistical models for yield prediction and genotype selection

Selecting appropriate statistical models is essential for improving the yield predictions in METs (Koundinya et al., 2021; Yue et al., 2022b). In this study, the Best Linear Unbiased Prediction (BLUP) model outperformed the Additive Main Effects and Multiplicative Interaction (AMMI) model for predicting yield. These findings support the use of BLUP as a preferable tool for large datasets, as it accounts for both genotype and environmental effects, and provides more reliable predictions for breeders (Piepho, 1994). While AMMI remains useful for modeling genotype–environment interactions and imputing missing data, our results suggest that BLUP is particularly valuable for predicting yield potential in trials with diverse environmental conditions. These insights can be directly applied to breeding programs that seek to optimize yield predictions and to select the best-performing genotypes for specific locations or climatic conditions (Piepho et al., 2008).

### 4.4 Application of GGE biplot for genotype evaluation and mega-environment identification

Wheat is a globally distributed and highly adaptable crop that not only has high nutritional value but also possesses unique gluten properties and excellent processing characteristics, making it suitable for the production of a wide variety of foods (Hyles et al., 2020; Yadav et al., 2022). Although China is the largest wheat producer in the world, with an annual production exceeding 137 million tons, accounting for approximately 17% of the global total, the mean yield in China is 5, 812 kg/ha, which is only approximately 80% of the yield levels seen in advanced agricultural countries like France. The analysis of numerous genotypes across diverse environments presents challenges in identifying consistent responses across environments, particularly without graphical representations of the data. The GGE biplot enabled visualization of genotype performance across multiple environments. By identifying mega-environments, the GGE biplot helps breeders focus on genotypes that are best suited to specific environmental conditions (Yan, 2001; Yan and Holland, 2010). Locations like Handan, Feixiang, Nanpi, and Shenzhou were consistently identified as representative environments over multiple years. These locations offer valuable insights for breeders looking to select genotypes that can thrive under specific climatic conditions, either for general or targeted adaptation. For example, genotypes such as JM196, WM4176, and ZX4899 performed consistently well in their respective mega-environments. These genotypes show promise for use in breeding programs that target specific regions. Moreover, environments with high discriminative power, such as Yongnian and Feixiang, are ideal for identifying stable genotypes with broad adaptability, contributing to the selection of high-yielding varieties suitable for various regions. Recognizing consistently similar environments aids in optimizing location selection for multi-environment trials (METs), thus reducing MET costs. Similar analyses have been applied to rice (Singh et al., 2023), maize (Yue et al., 2021), winter (Ferrante et al., 2021), and sugarcane (Mehareb et al., 2022), utilizing the GGE biplot to pinpoint the most discriminative test environments. This study also underscores the unpredictability of year-to-year variations at the same location, emphasizing the need for a stability analysis to better align specific genotypes with specific environments.

## 5 Conclusion

Breeding programs rely on multi-environment trials to identify the best-performing genotypes for commercial cultivation and to select locations that best represent the target environments. This study analyzed mega-environments from 2014 to 2018 using several statistical approaches: envirotyping, AMMI, BLUP, and GGE. The findings show that the AMMI index effectively identifies genotypes with minimal GEI, whereas the BLUP index is useful for selecting genotypes with high grain yield. In contrast, the WAASY index highlighted the genotypes that demonstrated superior performance in terms of both yield and stability. A positive correlation was observed between WAASY, grain yield, and BLUP indices. Principal component analysis (PCA) revealed that grain yield was positively correlated with environmental factors such as potential evapotranspiration (ETP), vapor pressure deficit (VPD), maximum air temperature (TMAX), and organic carbon (ORGC). Conversely, it was negatively correlated with relative humidity (RH2M), rainfall precipitation (PRECTOT), and dew-point temperature (T2MDEW). Interestingly, some geographically and agro-ecologically distinct locations exhibited similar data patterns and were grouped into the same mega-environment. This suggests that other biological, biophysical, and soil-related factors are important for classifying the environment. For breeding programs, it is recommended to select and test genotypes within the identified mega-environments to ensure that they are adapted to specific conditions. Locations such as Feixiang and Shenzhou were found to be both discriminative and representative, making them ideal locations for developing wheat cultivars with broader adaptability.

## Data Availability

The original contributions presented in the study are included in the article/**Supplementary Material**. Further inquiries can be directed to the corresponding authors.
