# Supplementary material for: Assessing the role of genotype by environment interaction of winter wheat cultivars using envirotyping techniques in North China
Source: Front Plant Sci. 2025 Feb 11;16:1538661. doi: 10.3389/fpls.2025.1538661 (PMC11850365; doi:10.3389/fpls.2025.1538661)
Supplement: Supplementary file 1 [file DataSheet1.pdf]

Supplementary Material

Supplementary Figures

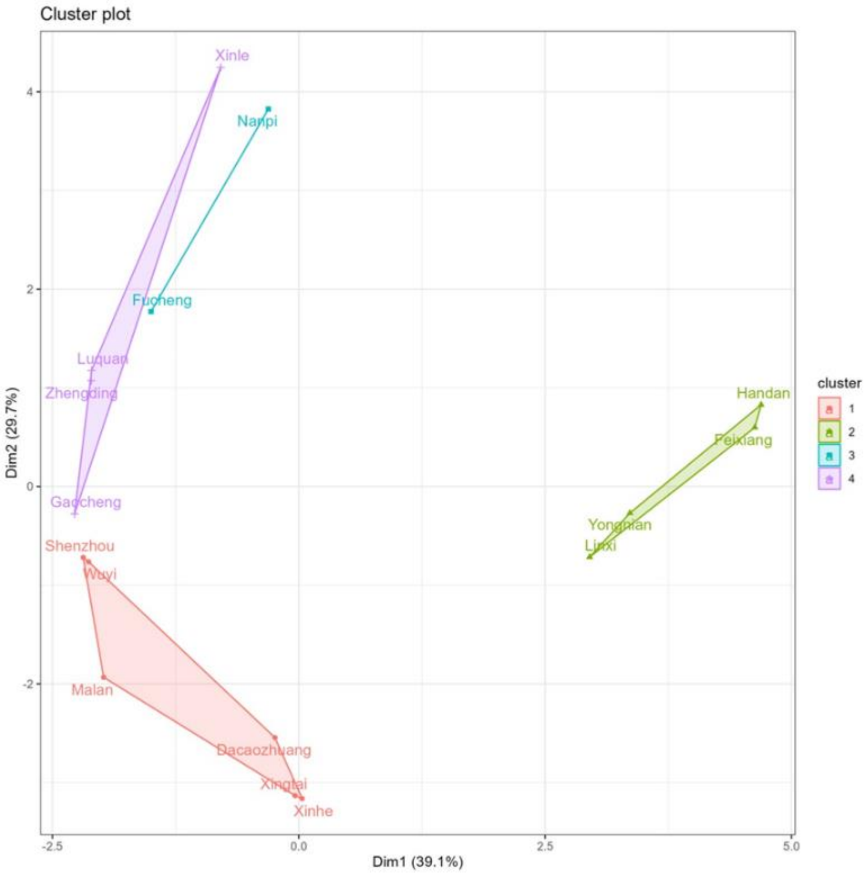

Figure S1. Four mega-environments cluster plot based on 30 years of climate information and soil covariates.

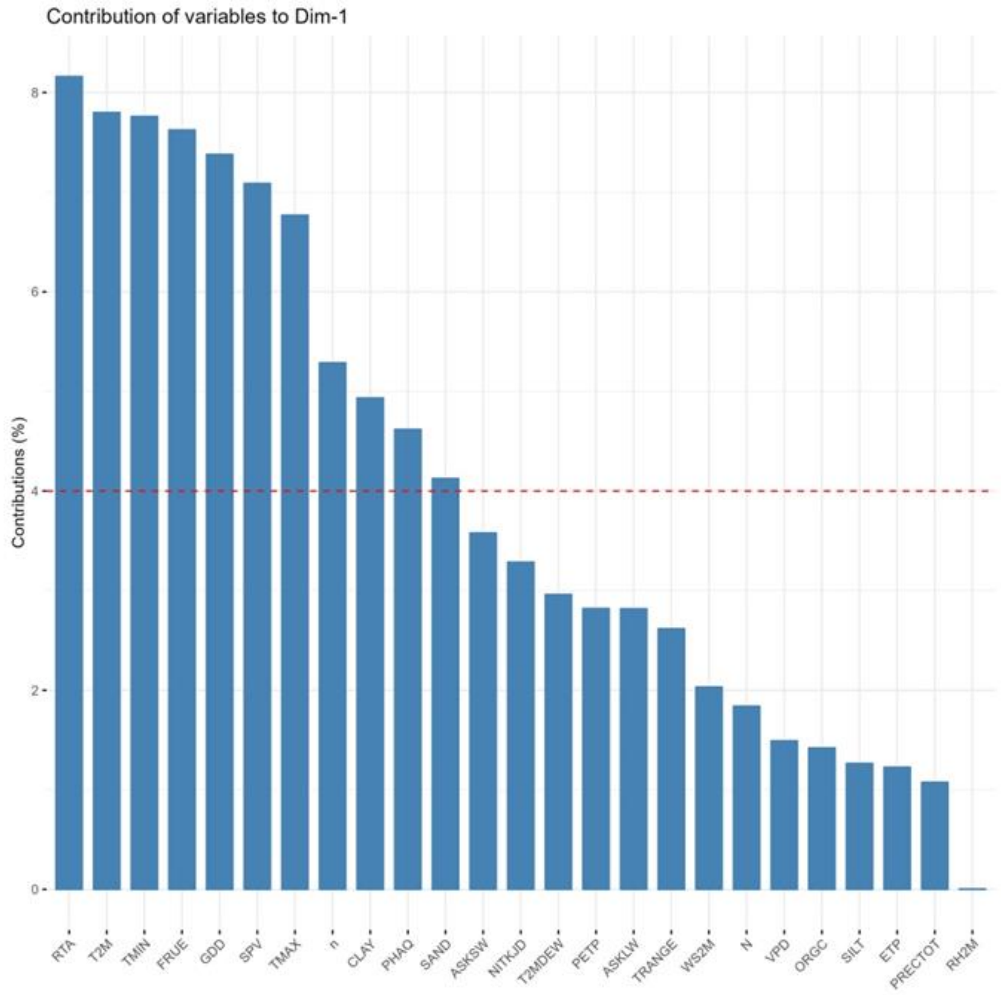

Figure S2. The contribution of environmental variables to the variation during 1989-2019.

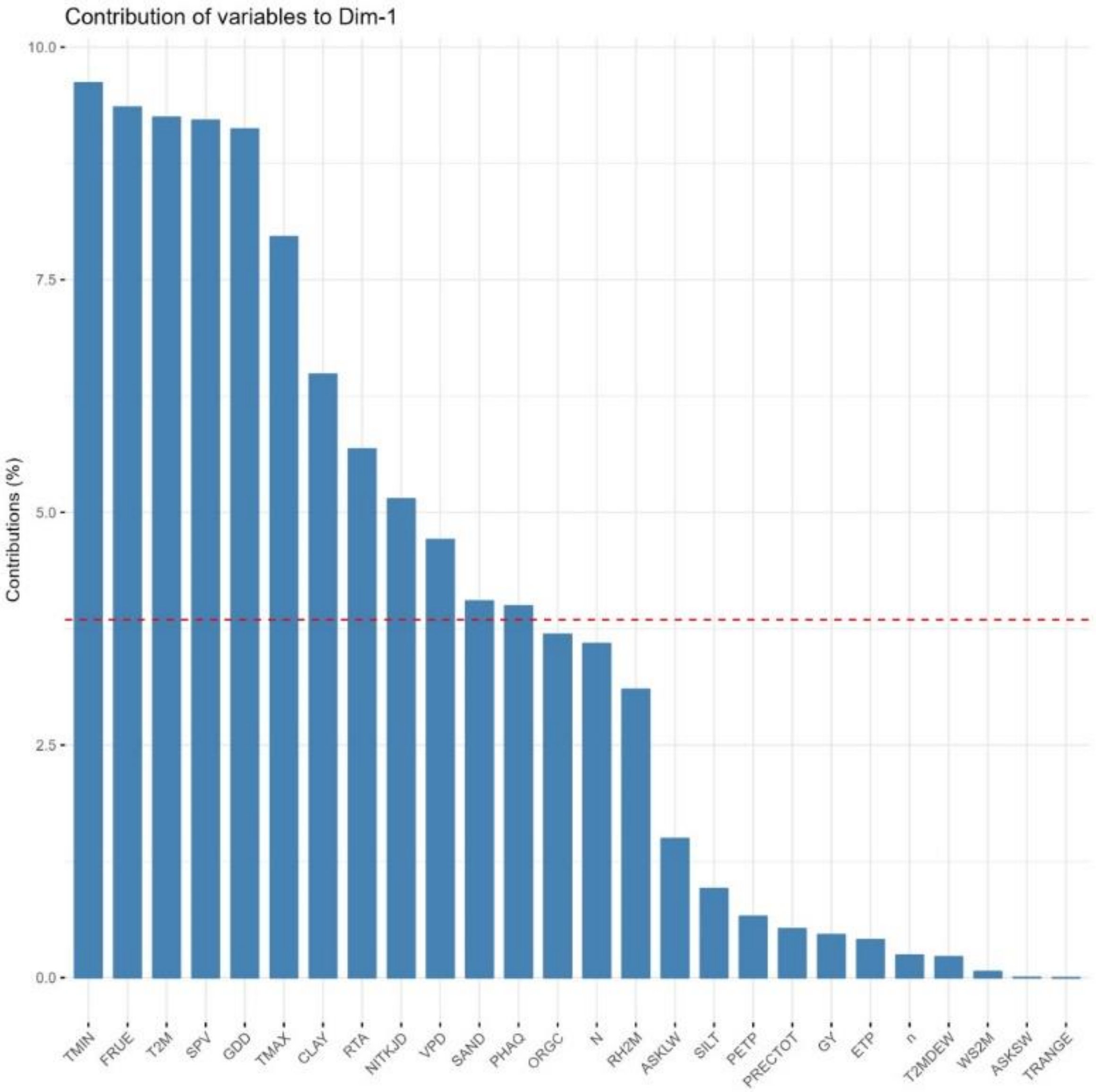

Figure S3. The contribution of environmental variables to the variation during 2014-2018.

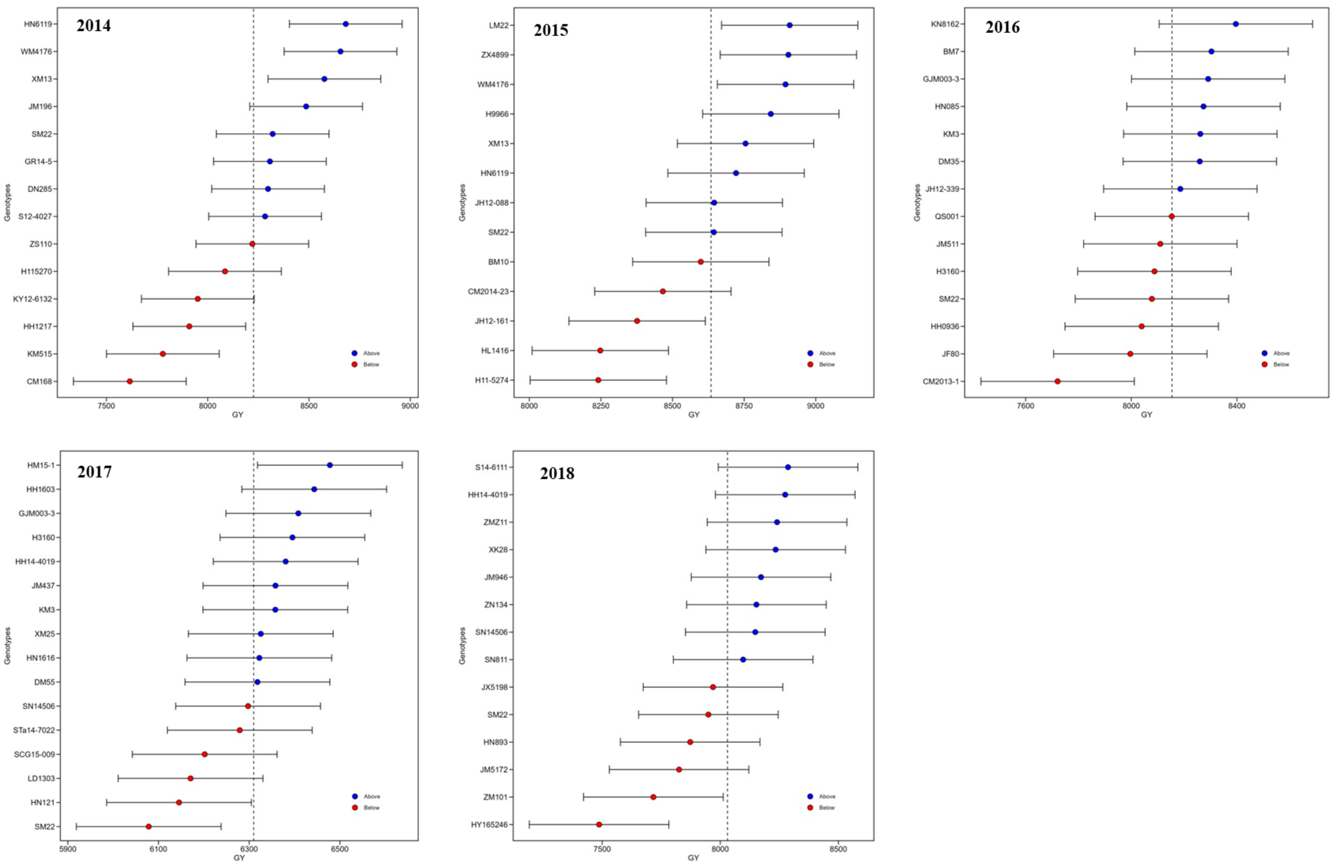

Figure S4. BLUP (Best linear unbiased predictor) values of winter wheat varieties for year 2014 to 2018.

Supplementary Tables

Table S1. Basic information of the tested genotypes during 2014.

Genotype	Code	Type of materials	Origin
GR14-15	14-1	Cultivar	China
CM168	14-2	Cultivar	China
DN285	14-3	Cultivar	China
H115270	14-4	Cultivar	China
HN6119	14-5	Breeding line	China
HH1217	14-6	Breeding line	China
JM196	14-7	Breeding line	China
KY12-6132	14-8	Breeding line	China
KM515	14-9	Breeding line	China
S12-4027	14-10	Breeding line	China
SM22	14-11	Breeding line	China
WM4176	14-12	Breeding line	China
XM13	14-13	Breeding line	China
ZS110	14-14	Breeding line	China

Table S2. Basic information of the tested genotypes during 2015.

Genotype	Code	Type of materials	Origin
CM10	15-1	Cultivar	China
CM2014-23	15-2	Cultivar	China
H11-5274	15-3	Cultivar	China
H9966	15-4	Cultivar	China
HL1416	15-5	Cultivar	China
HN6119	15-6	Cultivar	China
JH12-088	15-7	Cultivar	China
JH12-161	15-8	Cultivar	China
LM22	15-9	Breeding line	China
SM22	15-10	Breeding line	China
WM4176	15-11	Breeding line	China
XM13	15-12	Breeding line	China
ZX4899	15-13	Breeding line	China

Table S3. Basic information of the tested genotypes during 2016.

Genotype	Code	Type of materials	Origin
BM7	16-1	Cultivar	China
CM2013-1	16-2	Cultivar	China
DM35	16-3	Cultivar	China
GJM003-3	16-4	Cultivar	China
H3160	16-5	Cultivar	China
HN085	16-6	Cultivar	China
HH0936	16-7	Breeding line	China
JM511	16-8	Breeding line	China
JF80	16-9	Breeding line	China
JH12-339	16-10	Breeding line	China
KM3	16-11	Breeding line	China
KN8162	16-12	Breeding line	China
QS001	16-13	Breeding line	China
SM22	16-14	Breeding line	China

Table S4. Basic information of the tested genotypes during 2017.

Genotype	Code	Type of materials	Origin

DM55	17-1	Cultivar	China
GJM003-3	17-2	Cultivar	China
H3160	17-3	Breeding line	China
HN1616	17-4	Breeding line	China
HN121	17-5	Breeding line	China
HH14-4019	17-6	Breeding line	China
HH1603	17-7	Breeding line	China
HM15-1	17-8	Breeding line	China
JM437	17-9	Breeding line	China
KM3	17-10	Breeding line	China
LD1303	17-11	Breeding line	China
SCG15-009	17-12	Breeding line	China
STa14-7022	17-13	Breeding line	China
SM22	17-14	Breeding line	China
SN14506	17-15	Breeding line	China
XM25	17-16	Breeding line	China

Table S5. Basic information of the tested genotypes during 2018.

Genotype	Code	Type of materials	Origin
ZM101	18-1	Cultivar	China
HN893	18-2	Cultivar	China
HH14-4019	18-3	Cultivar	China
HY165246	18-4	Cultivar	China
JM5172	18-5	Cultivar	China
JM946	18-6	Cultivar	China
JX5198	18-7	Cultivar	China
SN811	18-8	Cultivar	China
S14-6111	18-9	Breeding line	China
SM22	18-10	Breeding line	China
SN14506	18-11	Breeding line	China
XK28	18-12	Breeding line	China
ZN134	18-13	Breeding line	China
ZMZ11	18-14	Breeding line	China

Table S6. Ranking of genotypes based on BLUP stability indices.

2014									
**Genotypes**	**GY**	**RPGV**	**RPGV_Y**	**RPGV_R**	**HMGV**	**HMGV_R**	**HMRPGV**	**HMRPGV_Y**	**HMRPGV_R**
CM168	7530	0.918	7555	14	7491	14	0.916	7533	14
DN285	8308	1.01	8292	8	8205	8	1.01	8274	8
GR14-5	8318	1.01	8310	7	8246	7	1.01	8306	6
H115270	8066	0.979	8052	10	7973	10	0.977	8039	10
HH1217	7865	0.958	7883	12	7828	12	0.957	7872	12
HN6119	8745	1.06	8720	1	8644	1	1.06	8711	1
JM196	8522	1.04	8525	4	8466	4	1.03	8513	4
KM515	7716	0.94	7733	13	7672	13	0.939	7721	13
KY12-6132	7912	0.962	7916	11	7844	11	0.96	7900	11
S12-4027	8291	1.01	8314	6	8264	5	1.01	8295	7
SM22	8334	1.01	8328	5	8263	6	1.01	8321	5
WM4176	8716	1.06	8703	2	8639	2	1.06	8695	2
XM13	8625	1.05	8616	3	8555	3	1.05	8609	3
ZS110	8220	0.999	8221	9	8165	9	0.999	8216	9

**2015**									
BM10	8592	0.994	8587	9	8472	9	0.994	8580	9
CM2014-23	8435	0.978	8448	10	8344	10	0.977	8439	10
H11-5274	8167	0.947	8175	13	8052	13	0.945	8164	13
H9966	8882	1.03	8886	4	8789	4	1.03	8875	4
HL1416	8175	0.951	8212	12	8123	12	0.95	8206	12
HN6119	8738	1.01	8732	6	8627	6	1.01	8730	6
JH12-088	8648	1	8631	8	8507	8	0.999	8624	8
JH12-161	8328	0.966	8341	11	8229	11	0.965	8331	11
LM22	8961	1.04	8944	1	8828	3	1.03	8932	2
SM22	8646	1	8652	7	8549	7	1	8645	7
WM4176	8943	1.03	8933	3	8832	2	1.03	8931	3
XM13	8778	1.02	8768	5	8661	5	1.02	8765	5
ZX4899	8955	1.04	8938	2	8832	1	1.03	8936	1
**2016**									
BM7	8382	1.02	8353	2	8184	2	1.02	8345	2
CM2013-1	7495	0.933	7606	14	7463	14	0.932	7598	14
DM35	8314	1.02	8291	5	8124	5	1.02	8287	5
GJM003-3	8363	1.02	8324	3	8151	3	1.02	8321	3
H3160	8053	0.994	8102	9	7963	9	0.992	8091	9
HH0936	7979	0.982	8007	12	7844	12	0.981	7998	12
HN085	8336	1.02	8306	4	8133	4	1.02	8298	4
JF80	7914	0.972	7927	13	7741	13	0.971	7918	13
JH12-339	8202	1.01	8205	7	8051	7	1.01	8203	7
JM511	8087	0.993	8095	10	7926	10	0.992	8087	10
KM3	8317	1.02	8277	6	8094	6	1.01	8271	6
KN8162	8522	1.04	8465	1	8285	1	1.04	8457	1
QS001	8153	1	8152	8	7987	8	1	8150	8
SM22	8038	0.987	8047	11	7879	11	0.987	8045	11
**2017**									
DM55	6321	1	6321	10	6254	10	1	6319	10
GJM003-3	6445	1.02	6439	3	6375	3	1.02	6437	3
H3160	6427	1.02	6413	4	6340	4	1.02	6411	4
HH14-4019	6407	1.01	6404	5	6337	5	1.01	6399	5
HH1603	6494	1.03	6482	2	6415	2	1.03	6477	2
HM15-1	6541	1.03	6519	1	6448	1	1.03	6518	1
HN121	6083	0.968	6110	15	6044	15	0.967	6103	15
HN1616	6327	1	6326	9	6257	9	1	6321	9
JM437	6376	1.01	6369	7	6299	7	1.01	6367	7
KM3	6376	1.01	6374	6	6309	6	1.01	6372	6
LD1303	6118	0.972	6135	14	6062	14	0.971	6127	14
SCG15-009	6161	0.978	6168	13	6091	13	0.977	6165	13
SM22	5991	0.953	6011	16	5930	16	0.951	6004	16
SN14506	6293	0.996	6287	11	6207	11	0.996	6282	11
STa14-7022	6268	0.994	6275	12	6207	12	0.994	6270	12
XM25	6331	1	6328	8	6258	8	1	6326	8
**2018**									

HH14-4019	8345	1.03	8311	2	8142	. 2	1.03	8305	2
HN893	7827	0.978	7855	11	7718	. 11	0.976	7842	11
HY165246	7331	0.921	7396	14	7270	. 14	0.918	7375	14
JM5172	7767	0.967	7765	12	7592	. 12	0.964	7745	12
JM946	8213	1.02	8204	5	8052	. 5	1.02	8198	5
JX5198	7952	0.989	7943	9	7780	. 9	0.988	7934	9
S14-6111	8360	1.04	8354	1	8190	. 1	1.04	8323	1
SM22	7926	0.986	7915	10	7754	. 10	0.985	7911	10
SN14506	8182	1.02	8171	7	8018	. 7	1.02	8166	7
SN811	8116	1.01	8119	8	7976	. 8	1.01	8113	8
XK28	8293	1.03	8263	4	8099	. 4	1.03	8261	4
ZM101	7627	0.955	7671	13	7543	. 13	0.954	7657	13
ZMZ11	8300	1.03	8277	3	8113	. 3	1.03	8271	3
ZN134	8188	1.02	8182	6	8035	. 6	1.02	8178	6

GY, grain yield (kg/ha); R, ranking of the given parameter; RPGV, the relative performance of the genotypic values; HMGV, the harmonic mean of genotypic values, HMRPGV, the harmonic mean of the relative performance of genotypic values.

Table S7. Ranking of genotypes based on AMMI and WAAS stability indices.

**2014**										
**Genotypes**	**Grain yield**	**ASTAB**	**ssiASTAB**	**ASI**	**ASI_SSI**	**ASV**	**ASV_SSI**	**AVAMGE**	**AVAMGE_SSI**	**DA**
CM168	7530	1350	28	7.06	27	33.1	27	4273	26	1714
DN285	8308	1335	20	3.22	12	15.1	12	4321	20	1535
GR14-5	8318	347	7	0.691	7	3.24	7	1855	7	665
H115270	8066	1224	22	6.79	22	31.9	22	3939	20	1551
HH1217	7865	725	19	3.34	21	15.7	21	3501	19	1203
HN6119	8745	711	7	6.19	11	29.1	11	2946	7	1229
JM196	8522	756	12	6.4	15	30.1	15	3658	13	1325
KM515	7716	1062	22	1.53	16	7.21	16	3550	21	1311
KY12-6132	7912	1090	21	3.24	17	15.2	17	4092	22	1454
S12-4027	8291	1189	19	7.58	22	35.6	22	4430	22	1654
SM22	8334	541	8	3.3	12	15.5	12	2249	8	977
WM4176	8716	661	7	3.14	6	14.7	6	2664	7	1043
XM13	8625	596	7	3.32	11	15.6	11	2656	7	998
ZS110	8220	479	11	1.45	11	6.8	11	2169	11	831
**Genotypes**	**DA_SSI**	**DZ**	**DZ_SSI**	**MASI**	**MASI_SSI**	**MASV**	**MASV_SSI**	**SIPC**	**WAAS**	**WAAS_SSI**
CM168	28	0.802	25	7.59	27	48.6	25	79.1	14.5	28
DN285	18	0.902	21	5.47	15	59.2	21	85.7	11.6	17
GR14-5	7	0.539	7	1.72	7	29.5	7	32	3.47	7
H115270	22	0.859	23	7.19	22	42.9	19	72.6	12	21
HH1217	18	0.631	17	4.91	19	39.1	20	50.3	8.38	19
HN6119	8	0.641	8	6.25	11	34.4	5	50.2	8.69	9
JM196	13	0.583	7	6.65	15	36.8	9	55.4	11.1	13
KM515	21	0.82	25	3.78	18	48.7	25	66.6	8.19	19
KY12-6132	21	0.778	21	5.78	20	51.3	24	77.2	12.1	23
S12-4027	21	0.737	17	8.08	22	47.3	18	69.8	14.5	21
SM22	8	0.58	7	3.62	8	31.4	7	49.8	6.51	8

WM4176	7	0.678	10	3.73	6	38.5	9	57.1	7.77	7
XM13	7	0.632	9	3.82	9	32	6	53.3	7.03	7
ZS110	11	0.592	13	2.27	11	38	15	42.7	4.97	11
	**2015**									
**Genotypes**	**Grain yield**	**ASTAB**	**ssiASTAB**	**ASI**	**ASI_SSI**	**ASV**	**ASV_SSI**	**AVAMGE**	**AVAMGE_SSI**	**DA**
BM10	8592	950	17	2.98	15	14.9	15	3547	17	1208
CM2014-23	8435	956	19	7.96	22	39.7	22	3343	16	1426
H11-5274	8167	1106	25	5.28	22	26.4	22	4029	24	1454
H9966	8882	988	14	7.83	15	39.1	15	3935	14	1467
HL1416	8175	870	19	2.81	17	14.1	17	3598	21	1208
HN6119	8738	340	9	1.34	9	6.7	9	1895	9	695
JH12-088	8648	752	13	4.98	15	24.9	15	3179	12	1183
JH12-161	8328	1102	22	6.16	21	30.7	21	4174	23	1442
LM22	8961	1382	14	9.4	14	46.9	14	4670	14	1755
SM22	8646	710	13	3.82	15	19.1	15	3459	15	1165
WM4176	8943	131	4	0.367	4	1.83	4	1187	4	419
XM13	8778	632	9	0.564	7	2.82	7	2118	9	874
ZX4899	8955	328	4	1.88	6	9.36	6	1544	4	678
**Genotypes**	**DA_SSI**	**DZ**	**DZ_SSI**	**MASI**	**MASI_SSI**	**MASV**	**MASV_SSI**	**SIPC**	**WAAS**	**WAAS_SSI**
BM10	17	0.811	22	4.42	14	42.9	17	71.2	10.6	8
CM2014-23	19	0.705	17	8.13	22	46.5	20	63.4	13.1	10
H11-5274	24	0.785	24	6.89	23	49	24	70.1	13.3	12
H9966	16	0.685	10	7.89	15	42.4	11	57.3	13.3	11
HL1416	19	0.736	21	4.58	18	43	21	67.4	10.5	7
HN6119	9	0.509	9	2.41	9	23	8	37.5	5.19	3
JH12-088	13	0.651	12	5.23	15	35.8	12	54.2	9.76	5
JH12-161	21	0.782	21	6.68	20	49.4	23	63	12.4	9
LM22	14	0.805	13	9.95	14	57.6	14	76.5	17.1	13
SM22	13	0.619	12	5.11	15	36.7	14	51.2	9.84	14
WM4176	4	0.321	4	1.27	4	14.1	4	21	2.65	4
XM13	9	0.731	13	2.07	7	28.7	9	45.7	4.61	7
ZX4899	4	0.503	4	2.42	6	23.3	5	40.2	5.52	6
	**2016**									
**Genotypes**	**Grain yield**	**ASTAB**	**ssiASTAB**	**ASI**	**ASI_SSI**	**ASV**	**ASV_SSI**	**AVAMGE**	**AVAMGE_SSI**	**DA**
BM7	8382	505	11	6.78	14	26.3	14	1936	9	1062
CM2013-1	7495	702	26	4.12	20	16	20	2726	26	1133
DM35	8314	361	12	4.55	13	17.6	13	2051	15	855
GJM003-3	8363	150	6	3.22	6	12.5	6	1363	7	561
H3160	8053	1070	24	9.48	24	36.8	24	3672	24	1530
HH0936	7979	508	22	6.58	23	25.5	23	2253	22	1056
HN085	8336	537	15	7.41	17	28.7	17	2297	15	1112
JF80	7914	409	20	5.36	21	20.8	21	1755	18	928
JH12-339	8202	231	11	3.69	11	14.3	11	1324	10	685
JM511	8087	736	22	3.71	14	14.4	14	2946	22	1150
KM3	8317	466	13	5.8	14	22.5	14	2050	13	992
KN8162	8522	352	6	5.89	11	22.8	11	1850	7	895

QS001	8153	52.7	9	2.17	10	8.44	10	795	10	343
SM22	8038	62.5	13	0.627	12	2.43	12	774	12	328
**Genotypes**	**DA_SSI**	**DZ**	**DZ_SSI**	**MASI**	**MASI_SSI**	**MASV**	**MASV_SSI**	**SIPC**	**WAAS**	**WAAS_SSI**
BM7	12	0.479	11	6.96	14	28.9	10	35.4	12.9	14
CM2013-1	26	0.624	26	5.58	21	30.8	24	42.2	12.5	24
DM35	11	0.425	12	4.83	11	26	12	31.7	10.4	11
GJM003-3	6	0.27	6	3.43	6	15.4	6	20.7	7.1	6
H3160	24	0.703	24	9.66	24	44.7	24	54.3	19.5	24
HH0936	21	0.484	22	6.69	23	30.8	23	37.3	13.4	25
HN085	15	0.485	15	7.51	17	30	13	33.2	12.6	15
JF80	20	0.444	20	5.59	21	27.3	20	34.6	11.9	22
JH12-339	11	0.339	11	3.9	11	20.9	11	25.3	8.32	11
JM511	22	0.643	22	5.44	15	31.2	21	41.1	11.6	17
KM3	13	0.471	13	5.8	14	32.2	18	29.4	10.5	11
KN8162	7	0.394	6	5.89	11	25.9	6	27.7	10.7	8
QS001	10	0.155	9	2.23	10	9.49	10	11.8	4.27	10
SM22	12	0.191	13	1.43	12	8.31	12	10.6	2.78	12
	**2017**									
**Genotypes**	**Grain yield**	**ASTAB**	**ssiASTAB**	**ASI**	**ASI_SSI**	**ASV**	**ASV_SSI**	**AVAMGE**	**AVAMGE_SSI**	**DA**
DM55	6321	28	11	1.27	11	5.81	11	539	11	199
GJM003-3	6445	53.9	5	1.44	5	6.58	5	621	5	265
H3160	6427	229	12	6.33	16	28.9	16	1252	11	655
HH14-4019	6407	358	16	8.06	20	36.8	20	1697	16	825
HH1603	6494	382	14	7.08	16	32.3	16	2055	15	805
HM15-1	6541	90.8	6	3.78	8	17.3	8	975	6	404
HN121	6083	680	31	6.21	26	28.3	26	2628	30	968
HN1616	6327	304	19	6.36	22	29	22	1641	19	720
JM437	6376	102	12	2.8	11	12.8	11	1003	12	386
KM3	6376	83.9	10	3.63	13	16.6	13	888	11	388
LD1303	6118	645	29	9.25	30	42.2	30	2737	30	1050
SCG15-009	6161	234	22	2.3	17	10.5	17	1375	21	541
SM22	5991	528	30	4.17	24	19	24	1713	28	828
SN14506	6293	191	18	5.36	20	24.4	20	1467	20	582
STa14-7022	6268	478	25	5.79	22	26.4	22	2216	26	835
XM25	6331	89	12	2.08	11	9.5	11	829	11	350
**Genotypes**	**DA_SSI**	**DZ**	**DZ_SSI**	**MASI**	**MASI_SSI**	**MASV**	**MASV_SSI**	**SIPC**	**WAAS**	**WAAS_SSI**
DM55	11	0.142	11	1.31	11	7.79	11	8.43	2.6	11
GJM003-3	5	0.207	5	1.73	5	9.4	5	11.6	3.44	6
H3160	13	0.352	12	6.34	15	29.4	13	21.4	9.44	13
HH14-4019	17	0.438	16	8.09	20	37.2	20	25.6	11.5	18
HH1603	13	0.484	14	7.16	16	34.7	15	32.3	12.3	16
HM15-1	7	0.229	5	3.82	8	17.8	7	14.5	5.94	8
HN121	30	0.723	31	7.03	28	35.9	29	38.7	12.5	30
HN1616	19	0.431	18	6.43	21	31.1	20	28.7	11	20

JM437	10	0.271	12	3.01	11	15.1	10	16.8	5.61	12
KM3	12	0.221	10	3.68	13	17	12	12.6	5.32	11
LD1303	30	0.624	28	9.26	30	45.7	30	38.1	15.4	30
SCG15-009	20	0.434	23	2.86	17	18.9	20	22.5	5.36	18
SM22	29	0.644	31	4.8	24	29.9	26	36.9	10.1	26
SN14506	19	0.333	18	5.38	20	25.6	19	21.7	8.76	19
STa14-7022	26	0.578	25	5.86	22	33.6	24	34.1	11.2	24
XM25	11	0.254	13	2.08	11	13.7	11	10.2	2.93	10
	**2018**									
**Genotypes**	**Grain yield**	**ASTAB**	**ssiASTAB**	**ASI**	**ASI_SSI**	**ASV**	**ASV_SSI**	**AVAMGE**	**AVAMGE_SSI**	**DA**
HH14-4019	8345	358	8	4.38	7	21.3	7	2486	9	868
HN893	7827	666	21	13	22	63	22	3558	22	1474
HY165246	7331	1188	27	13.9	27	67.7	27	4687	28	1841
JM5172	7767	950	24	13.3	24	64.6	24	3940	24	1630
JM946	8213	291	8	5.9	11	28.7	11	1754	8	808
JX5198	7952	683	20	4.1	12	19.9	12	2673	18	1064
S14-6111	8360	1900	15	16.7	15	81.1	15	4051	14	2252
SM22	7926	156	11	4.18	14	20.3	14	1641	12	627
SN14506	8182	346	12	5.95	14	28.9	14	2350	13	883
SN811	8116	461	16	7.41	16	36	16	2342	13	1041
XK28	8293	308	8	3.67	6	17.8	6	1862	8	716
ZM101	7627	610	22	12.2	23	59.4	23	3386	23	1413
ZMZ11	8300	367	10	8.16	12	39.7	12	2546	11	1011
ZN134	8188	276	8	2.02	7	9.8	7	1363	7	664
**Genotypes**	**DA_SSI**	**DZ**	**DZ_SSI**	**MASI**	**MASI_SSI**	**MASV**	**MASV_SSI**	**SIPC**	**WAAS**	**WAAS_SSI**
HH14-4019	7	0.433	8	4.43	7	44.2	9	32.7	7.74	6
HN893	22	0.487	20	13	22	64.4	22	39.5	15.9	21
HY165246	27	0.677	27	13.9	27	89.8	27	57.3	20.6	27
JM5172	24	0.648	23	13.4	24	70.7	24	56.3	18.4	24
JM946	9	0.406	8	5.97	11	32.7	9	31.8	8.57	10
JX5198	18	0.67	21	4.43	13	44.3	17	49.1	8.87	15
S14-6111	15	0.907	15	16.7	15	107	15	82.1	25.7	15
SM22	11	0.263	11	4.2	13	31.3	13	22.3	6.73	13
SN14506	13	0.429	11	6.03	14	39	12	36.2	9.68	14
SN811	16	0.492	18	7.47	16	46.3	17	38.3	11.3	16
XK28	7	0.462	12	3.8	6	27.1	5	29.7	6.42	6
ZM101	23	0.456	20	12.2	23	63.6	23	39.9	16.1	24
ZMZ11	10	0.402	5	8.2	12	44.1	9	34	11.4	12
ZN134	8	0.43	11	2.22	7	29	8	28.4	4.08	7

GY, grain yield (kg/ha); ASTB, AMMI based stability parameter; ASI, AMMI stability index; ASV, AMMI-stability value; AVAMGE, sum across environments of absolute value of GEI modelled by AMMI; DA, Annicchiarico's D parameter values; DZ, Zhang's D parameter; MASI, modified AMMI stability index; MASV, modified AMMI-stability value; SIPC, sums of the absolute value of the IPC scores; WAAS, weighted average of absolute scores; SSI, simultaneous selection indexes.
